# Evaluation of joint and muscle function of paediatric upper and lower extremities

**DOI:** 10.3389/fbioe.2025.1667865

**Published:** 2025-10-29

**Authors:** Mohamed Afifi, Muhammad Uba Abdulazeez, Kamiar Aminian, Georgios Antoniou Stylianides, Kassim Abdulrahman Abdullah

**Affiliations:** ^1^ Department of Mechanical and Aerospace Engineering, College of Engineering, United Arab Emirates University, Al Ain, United Arab Emirates; ^2^ Emirates Centre for Mobility Research, United Arab Emirates University, Al Ain, United Arab Emirates; ^3^ Department of Automotive Engineering, Faculty of Engineering and Engineering Technology, Abubakar Tafawa Balewa University, Bauchi, Nigeria; ^4^ Laboratory of Movement Analysis and Measurement, École Polytechnique Fédérale de Lausanne, Lausanne, Switzerland; ^5^ Exercise Science and Kinesiology, Juniata College, Huntingdon, PA, United States

**Keywords:** muscle strength, joint stiffness, range of motion, extremity joints, children

## Abstract

Road traffic crashes are the leading cause of injuries, disabilities, and fatalities for children and young adults. Extremity joint injuries have been identified as one of the contributing factors to chronic disabilities among children in road crashes. However, our knowledge on the biomechanics of the pediatric upper and lower extremity joints remains limited. Understanding the biomechanics of the upper and lower extremity joints is essential to provide important information for developing enhanced protection against extremity joint injuries for children involved in road crashes. The protocol developed in this study will be used for assessing the following biomechanical properties of the pediatric upper and lower extremity joints: 1) active and passive ranges of motion (AROM and PROM), 2) muscle strength, and 3) joint stiffness. The joints included in the protocol are shoulder, elbow, wrist, hip, knee and ankle. Joint-specific settings and testing procedures are provided for assessing the range of motion (ROM) using goniometry and the muscle strength as well as joint stiffness using isokinetic dynamometry. A sample of 200 healthy children will be recruited from selected schools in Al Ain city, United Arab Emirates for the assessment. Descriptive statistical analyses will be conducted to characterize the biomechanical properties with regards to age, gender, and ethnicity. To determine the influence of anthropometric and demographic factors on ROM, strength, and stiffness, a series of multiple regression analyses will be performed to identify the factors that best predict ROM, strength, and stiffness.

## 1 Introduction

Recent advancements in technology have greatly enhanced our ability to analyse the biomechanics of extremity joints. This progress has led to the development of targeted training programs aimed at improving motor performance and developing measures to reduce relative risks of injury and implementing appropriate clinical interventions (De Ste Croix and Korff, 2013). The World Health Organization reported that road traffic crashes are the leading cause of injuries, disabilities, and fatalities for children and young adults aged 5–29 years (Organization, 2018). The head is the most frequently injured body region in children due to motor vehicle crashes worldwide and head injuries results in more fatalities compared to injuries in other parts of the child’s body (Hanna, 2010; WHO, 2007). However, recent advancements in vehicle safety and child restraints have significantly helped in reducing these injuries and fatalities (Seidel et al., 2018; Boucher et al., 2016). On the other hand, injuries to both the lower and upper extremities of the child’s body are the second most frequent injuries sustained by children in vehicle crashes worldwide (Hanna, 2010; Jermakian et al., 2007; Fildes et al., 1997). These injuries are usually not life threatening but mostly results in long-term suffering and even disabilities in some cases (Fildes et al., 1997; Boucher, 2014).

Several studies have been conducted on the biomechanics of the vulnerable parts of the child’s body susceptible to injuries in vehicle crashes including the head, chest, abdomen, pelvis, neck, and spine (Jiang et al., 2014; Luck et al., 2013; Margulies and Thibault, 2000; Ouyang et al., 2005; Arbogast and Maltese, 2014). Additionally, numerous studies have been performed regarding paediatric extremity bone biomechanical tolerance (Rockwood, 2010; Miltner and Kallieris, 1989; Chung et al., 1976; Currey and Butler, 1975; Ouyang et al., 2003a; Ouyang et al., 2003b). Furthermore, majority of the literature on extremity injuries in children due to vehicle crashes are focused on bone fractures (Hanna, 2010; Jermakian et al., 2007; Fildes et al., 1997; Arbogast et al., 2003; Bennett et al., 2006). On the other hand, upper extremity joint dislocation has been identified as one of the factors contributing to long-term disability in children due to contact with deploying airbags (Jernigan et al., 2005). Ankle joint kinematics and stiffness have also been found to contribute to ankle and foot fractures and subsequent injury severity (Seidel et al., 2018; Fildes et al., 1997; Boucher et al., 2017). Therefore, studying the biomechanics of the upper and lower extremity joints is essential to provide important information for developing enhanced protection against extremity injuries in children involved in vehicle crashes. Furthermore, the data collected in the proposed study will be valuable to the medical field for diagnosing and treating extremity injuries in children resulting not only from vehicle crashes but also injuries from sports and falls. It will also aid in the assessment and rehabilitation of children with disabilities such as autism, cerebral palsy (CP), and Down syndrome (DS).

This protocol is being developed to help conduct extensive assessments of the pediatric extremity joint function for the upper and lower extremities joints (shoulder, elbow, wrist, hip, knee, and ankle). The protocol will focus on the assessment of the joints’ range of motion (ROM) and stiffness as well as the strength of the muscles surrounding these joints. These biomechanical properties have been recognized as important factors in determining children’s biomechanical tolerance to vehicle-related crash impact (Seidel et al., 2018; Fildes et al., 1997; Boucher et al., 2017). Evaluating these properties will provide a better understanding of the children’s extremity joints’ response to crash forces thereby contributing to the development of more biofidelic pediatric computational models and crash test dummies. This will lead to the development of more effective child vehicle safety systems (i.e., child restraint devices).

ROM measurement is a common clinical assessment among adults and children. It indicates the maximum angular displacement a joint can move between the flexed position and the extended position (Goonetilleke and Karwowski, 2017). The motion resulting in the angular displacement of a joint can be performed either actively or passively. Active ROM (AROM) involves the movement produced by an individual’s voluntary unassisted muscle contractions, while passive ROM (PROM) refers to the motion produced by the application of an external force by an examiner (Norkin and White, 2016). Different instruments have been adopted to measure the ROM such as universal goniometers, electro-goniometers, inclinometers, optical motion capture (OMC) systems, smartphone applications, etc. (Ore et al., 2020; Krause et al., 2013; Keogh et al., 2019; Özüdoğru et al., 2024). In the proposed study, the AROM and PROM of the upper and lower extremities will be measured using goniometers and an inclinometer. The application of these instruments will be further discussed in Section 2.2 (Methodology). Although OMC is considered the gold standard for kinematic measurements (Ore et al., 2020), it is conducted in laboratories using high precision cameras and typically involves small sample sizes (Zhang et al., 2013). The use of a goniometer however, is easy to handle and time effective especially with larger sample sizes (Fraeulin et al., 2020).

The term “muscle function” is often used to describe different aspects of the muscle such as strength and power and its physical fitness. Muscle strength refers to the maximum force a muscle or group of muscles can exert during voluntary movement under specified testing conditions. Power is derived from the product of the force created and the velocity of the movement (Jones and Stratton, 2000). Muscle strength has been evaluated using different methods such as manual tests (Marcolin et al., 2009), hand-held dynamometry (van den Beld et al., 2006), and isokinetic dynamometry (Muñoz-Bermejo et al., 2019; Tsiros et al., 2011; Santos et al., 2013). Muscle function can be evaluated under certain conditions such as isometric, isotonic and isokinetic contractions. Isometric testing refers to the measurement of muscle tension produced at a specific point in the ROM under static conditions, i.e., variable effort to produce a force against an immovable object (Jones and Stratton, 2000). Isotonic testing simply involves movement about the joint axis where the resistance to movement is constant, but the velocity is variable, i.e., lifting an object with a particular weight. Isotonic testing often represents everyday functional activities (Jones and Stratton, 2000). Isokinetic testing, however, involves movement at a constant angular velocity about a joint axis with variable resistance. Isokinetic testing typically requires a device (isokinetic dynamometer) to control the velocity of the motion while applying the force necessary to maintain that velocity (Jones and Stratton, 2000). Both isotonic and isokinetic testing may involve concentric (shortening) or eccentric (lengthening) muscle contractions. Eccentric muscle testing is generally regarded as more stressful on the body as it requires more effort to oppose the resistance acting upon the muscle, therefore it is more likely to result in faster muscle discomfort (Jones and Stratton, 2000). However, the likelihood of muscle injuries during eccentric contractions is minimal provided that the subjects are given adequate warm-up and familiarization (De Ste Croix and Korff, 2013).

From a biomechanical viewpoint, joint stiffness determines how easily a joint can be manoeuvred by the surrounding muscles (Vigotsky et al., 2020). In order to measure stiffness of the muscles and joints, it is important to redefine the notion of stiffness and clarify how it can be measured in an experiment (Latash and Zatsiorsky, 1993). Farley et al. (1998) defined joint stiffness as the change in joint moment *M*, divided by the change in the joint angle *α*, denoted by Equation 1 below:

$$K_{joint}=\frac{dM}{d\alpha }$$ (1)

The muscle force-stiffness relationship is generally modelled as linear, but the process is highly dynamic requiring a coordinated interaction between the muscles supporting the joint (Brown and McGill, 2005). Latash and Zatsiorsky signified true stiffness of the human joint as the combination of all the individual stiffness values contributed by muscles, tendons, ligaments, cartilages, and bones. Therefore, they introduced the term “quasi-stiffness” to represent the collective behavior of the aforementioned components in a single value (Latash and Zatsiorsky, 1993). However, the mathematical expressions to model all the components that contribute to a certain motion have not been developed yet (Butler et al., 2003). Furthermore, quasi-stiffness cannot be assessed when the system is static as there is no change in the joint angle (Vigotsky et al., 2020).

Joint stiffness has also been defined as the resistance to displacement within a given joint at a specific angle (Struzik et al., 2021; Serpell et al., 2012). For the purpose of this study, only the quasi-stiffness (which will be referred to as dynamic stiffness) will be assessed. In essence, stiffness may be calculated in several ways depending on the research objective and the resources available. This study will focus on the stiffness at the joint level; hence the torsional stiffness will be examined. The methods applied to measure stiffness should be defined and comparisons between studies using different methods should be performed while considering the fact that different methods will likely produce different results (Butler et al., 2003).

While it is important to measure the muscle strength during different types of contractions, the protocol presented will highlight an approach to measure the isokinetic strength of the muscles because isokinetic strength measurements show higher reliability (Danneskiold‐Samsøe et al., 2009). To measure the muscle strength, the speed controlling mechanism in the dynamometer sets the maximum achievable velocity of the moving limb, so any additional force exerted by the participant is absorbed into the device and transformed into increased resistance. Therefore, the benefit of isokinetic muscle strength testing is that the measurement gives information about the dynamic qualities of the muscle tested, as it best mimics real-life scenarios in a safe and controlled process (Danneskiold‐Samsøe et al., 2009; Osternig, 1986). When measuring the dynamic stiffness of the involved joints, the dynamometer offers a reactive mode of operation, i.e., the lever arm of the device is set to move at a selected angular velocity which does not rely on the active muscle contraction, but rather the reactive muscle tension. The muscle strength and joint stiffness will be measured using the Biodex isokinetic dynamometer (Biodex System 4; Biodex Medical Systems Inc., New York). Generally, the isokinetic dynamometer offers a safe and controlled environment to examine muscle function (Gleeson and Mercer, 1996). A study by Tsiros et al. (2011) provided a critical evaluation of the Biodex isokinetic dynamometer for knee strength testing in children. Information regarding the reliability and safety of the device will be considered when performing the tests.

To the best of our knowledge, only a limited number of studies have assessed muscle strength and joint stiffness in pediatric extremity joints, with most of the studies focusing on children with disabilities such as cerebral palsy and Down syndrome. Additionally, these studies primarily examined lower extremity joints only, with particular emphasis on the knee joint (Muñoz-Bermejo et al., 2019; Tsiros et al., 2011; Saarinen et al., 2008; Haberfehlner et al., 2015; Ferreira et al., 2020). While much of the existing research has focused on adult populations, there is a growing body of work emphasizing the importance of extremity joint assessments in both healthy and pathological pediatric populations (Saarinen et al., 2008; Haberfehlner et al., 2015; Ferreira et al., 2020). However, the studies on the biomechanical properties of pediatric extremities often examine one or two properties of a single joint only. Therefore, the aim of this study is to develop a protocol using goniometry and isokinetic dynamometry to 1) measure the AROM and PROM of the paediatric upper and lower extremity joints, 2) evaluate the dynamic stiffness of the paediatric upper and lower extremity joints, and 3) assess the isokinetic strength of the muscles surrounding these joints.

## 2 Materials and equipment

### 2.1 Participants

The guideline provided by ISO 15535 (ISO, 2008) standard was used to establish the sample size required for this study according to Equation 2 below:

$$n={\left(\frac{1.96\times CV}{a}\right)}^{2}\times 1.534^{2}$$ (2)

where 1.96 is the critical value from a standard normal distribution for a 95% confidence interval; CV is the coefficient of variation; and *a* is the proportion of relative certainty required. Based on the values of CV provided by Pheasant and Halslegrave (Pheasant and Haslegrave, 2016), the sample size was obtained as follows:

$$n={\left(\frac{1.96\times 13}{2}\right)}^{2}\times 1.534^{2}=190.97=191\;subjects$$

Therefore, a sample of 200 healthy children (100 boys and 100 girls) will be recruited from selected schools in Al Ain city, Abu Dhabi, United Arab Emirates. Ethical approval for the study has been obtained from the United Arab Emirates University’s (UAEU) Human Ethics Research Committee (ERH_2022_1306_15). The inclusion criteria for this study are: 1) children aged 4–12 years; and 2) absence of any reported medical conditions such as musculoskeletal, neurological, neuromuscular, or rheumatic disorders. Conversely, children with impairments that may limit the normal joint ROM will be excluded. Normal joint ROM will be determined by the end-feels, which is defined as the barrier to further joint motion detected by the examiner (Norkin and White, 2016). Abnormal end-feels are considerably more painful than normal end-feels (Hayes and Petersen, 2001). To ensure an accurate representation of the child population, the subjects will be recruited based on their ethnicity, age, and gender to guarantee a stratified sampling based on these criteria. Demographic information about the participants, including age, gender and ethnicity will be collected before commencing the measurement process.

Children aged between 4 and 12 years are eligible to participate in the study. Age-specific inclusion criteria include children being able to participate in age-appropriate activities of daily living and being healthy for their age. Children that are unable to follow age-appropriate instructions will be excluded from the study. Twenty two children per year from 4 to 12 years will be recruited. Parental written informed consent and the children’s assent will be obtained before conducting the measurement process. The children will be recruited from the different schools in Al Ain city that have agreed to participate in the study. The principal investigator (last author) together with the researchers involved in the study (first and second authors) will explain the study to the school authorities and the contact person (classroom teacher, school nurse, physical education teacher, social worker) in each school. Thereafter, these contact persons will convey the details of the study to both the children and their parents. Due to the different arrangements within these schools, the children will be recruited through either of the four channels mentioned above. Flyers containing the details of the study together with the informed consent forms will be distributed to the children for onward delivery to their parents through the different channels. Additionally, details regarding the study will be shared directly to the parents through the social media platforms of the respective schools. The children that verbally assented to be involved in the study and whose parents consented for them to participate (by filling and returning the distributed informed consent forms) will be recruited. Finally, only the children that fulfilled the eligibility criteria for the study will be selected to participate.

### 2.2 Anthropometric measurements

Several anthropometric measurements will be recorded prior to the ROM, muscle strength and joint stiffness data collection. The children’s height and body mass will be measured using a stadiometer (Seca, Hamburg, Germany) and weighing scale (Seca, Hamburg, Germany) respectively while their waist circumference will be measured using a measuring tape (Seca, Hamburg, Germany). An anthropometer (Seca, Hamburg, Germany) will be used to measure the bideltoid breadth, biacromial breadth, bi-trochanter breadth, buttock-knee length, knee height and functional leg length. A segmometer (Cescorf, Porto Alegre, Brazil) will be used to measure the shoulder-to-elbow length, elbow-to-hand length and foot length.

### 2.3 ROM

The AROM and PROM of the upper and lower extremities will be measured using: 1) universal goniometer (Baseline Fabrication Enterprises, New York, USA), 2) electronic goniometer (Vernier, Beaverton, United States) and 3) inclinometer (Baseline Fabrication Enterprises, New York, United States). A universal goniometer will be employed for joints where positioning it along the anatomical landmarks is feasible, i.e., measuring the ROM of the wrist flexion by aligning the goniometer with the ulna and the little finger. The electronic goniometer (rotary potentiometer), in particular, will be used for joints where the goniometer straps can be securely attached, offering more precise measurements and reducing human reading errors where possible. For rotations where the goniometer cannot be properly aligned with anatomical landmarks, such as the internal and external rotations of the knee, the inclinometer (with an internal bubble indicating the angle of inclination) will be utilized. It is important that no contraindications to the joint rotations exist during the ROM assessment, such as pain due to an inflammation, or after an injury where there has been disruption of soft tissue (i.e., muscle, tendon, ligament, etc.) (Clarkson, 2020). ROM measurements will be performed in accordance with the standardized guidelines outlined by Norkin and White (Norkin and White, 2016). In addition, a trained researcher (the first author) will be conducting the measurements while another trained researcher (the second author) will be recording the data to ensure consistency across all the measurements. A similar approach will be employed for the strength and stiffness assessments to eliminate the issue of interrater variability. Standardization procedures outlined by Biodex such as participant positioning, stabilization, alignment with anatomical landmarks, and consistent verbal instructions will be strictly followed throughout the data collection process.

### 2.4 Isokinetic dynamometry

The muscle strength and joint stiffness will be measured using the Biodex System 4 dynamometer available at the Biomechanics Lab, UAEU. For consistency and clarity, guidelines and protocols outlined in the Biodex manual will be followed to ensure proper usage and accurate measurements. Muscle strength will be obtained directly from the dynamometer, while the stiffness will be calculated as the average gradient of the torque-angle curve (Bressel et al., 2004).

## 3 Methods

This protocol describes a cross-sectional study aimed at establishing reference values for joint ROM, muscle strength, and joint stiffness. The ROM, muscle strength and joint stiffness measurements will be conducted over 6 days for each subject, with each day allotted to a specific joint (Day 1 – Shoulder, Day 2 – Elbow, Day 3 – Wrist, Day 4 – Hip, Day 5 – Knee, and Day 6 – Ankle), testing 10 subjects per day (See Table 1). All measurements will be performed bilaterally, assessing both dominant and non-dominant limbs. Upper and lower limb dominance will be defined by the participant’s preferred side for throwing and kicking a ball (Ty Hopkins et al., 2007; Richards et al., 2016; Barnes et al., 2001). The potential psychological and physical burden associated with multi-day measurements will be carefully managed by assessing one joint per day. Testing sessions will be scheduled with sufficient rest between days, limited in duration to accommodate the children’s attention span, and will be conducted in a supportive child-friendly manner. Verbal encouragement, breaks, and small incentives (e.g., snacks, fun certificates, toys, and gift cards as appropriate) will be provided to maintain motivation. It will be emphasized that participation will remain voluntary, with the option to withdraw at any time. In addition, the children will perform three repetitions of isokinetic contractions at maximal effort for each joint motion as it has been recommended for adults that two to six repetitions are sufficient to obtain maximal values without resulting in muscle fatigue (Osternig, 1986). Moreover, the velocities set in this protocol will fall below 120°/sec as it is difficult for children to attain velocities beyond this limit (Docherty, 1996). Although the estimated timeline for data collection is approximately 20 weeks, we anticipate that the process may extend over the course of a year due to the possibility of interruptions such as subject availability, scheduling conflicts or any unforeseen delays.

**TABLE 1:** Multi-day measurement schedule.

Day	Joint	Movement
1	Shoulder	Flexion/Extension Abduction/Adduction Internal/External Rotation
2	Elbow	Flexion/Extension Forearm Pronation/Supination
3	Wrist	Flexion/Extension Ulnar/Radial Deviation
4	Hip	Flexion/Extension Abduction/Adduction
5	Knee	Flexion/Extension
6	Ankle	Plantarflexion/Dorsiflexion Inversion/Eversion

### 3.1 ROM measurement

Goniometry measurements will follow the standard guidelines provided by Norkin and White (2016) and Clarkson (2020). Tables 2, 3 provide a summary of the process for the ROM measurements.

**TABLE 2:** Upper extremity joints ROM measurement procedures.

Motion		Starting position	Procedure
Shoulder	Flexion/Extension^a^	The subject will be seated with his/her arms by the side and palm facing medially (see Figure 1A)	The shoulder will be flexed by moving the humerus in an anterior and upward direction. The shoulder will be extended by moving the humerus in the posterior direction until the limit of motion
	Abduction/Adduction^a^	The subject will be seated with his/her arms by the side and palm facing anteriorly. Maximum adduction will be recorded as the starting position for shoulder abduction (see Figure 1B)	The shoulder will be abducted by moving the arm laterally while maintaining lateral rotation and neutral extension/flexion
	Internal/External Rotation^a^	The subject will be positioned supine on a table with the shoulder abducted to 90° and elbow flexed to 90°. The upper arm will be supported by the table (see Figure 1C)	The shoulder will be medially rotated by moving the forearm downward causing the palm of the hand to face the floor. The shoulder will be laterally rotated by moving the forearm downward causing the dorsal surface of the hand to face the floor. Shoulder abduction and elbow flexion will be maintained during this motion
Elbow	Flexion/Extension^b^	The subject will be seated with his/her arms fully extended by the side of the body and his/her palm facing anteriorly (see Figure 2A)	The elbow will be flexed by moving the hand upward toward the shoulder while maintaining forearm supination. The elbow will be extended/hyperextended by moving the hand in the posterior direction to the limit of the motion while maintaining forearm supination
	Pronation/Supination of the forearm^a^	The subject will be placed in the seated position with the shoulder in 0° flexion and abduction, and elbow flexed to 90°. The forearm will be positioned midway between supination and pronation such that the thumb is pointing upwards. The subject will tightly grip a pencil in a closed fist, with the pencil extending from the radial side of the hand to facilitate accurate measurements (see Figure 2B)	The forearm will be pronated by rotating the hand such that the palm faces downwards. The forearm will be supinated by rotating the hand such that the palm faces upwards
Wrist	Flexion/Extension^a^	The subject will be positioned seated next to a supporting surface with the elbow at 90° flexion and shoulder abducted to rest the forearm on the supporting surface. The forearm will be pronated so the palm faces the floor (see Figure 3)	The wrist will be flexed by moving the hand downward, so the palm faces posteriorly. The wrist will be extended by moving the hand upward so that the dorsal surface of the hand faces posteriorly. The wrist will be held at 0° of ulnar and radial deviation during both motions
	Ulnar/Radial Deviation^a^		The wrist will be radially deviated by moving the hand towards the thumb. The wrist will be deviated in the ulnar direction by moving the hand towards the little finger. The wrist will be maintained at 0° of flexion and extension during both motions

^a^ Universal goniometer.

^b^ Electronic goniometer.

**FIGURE 1:**
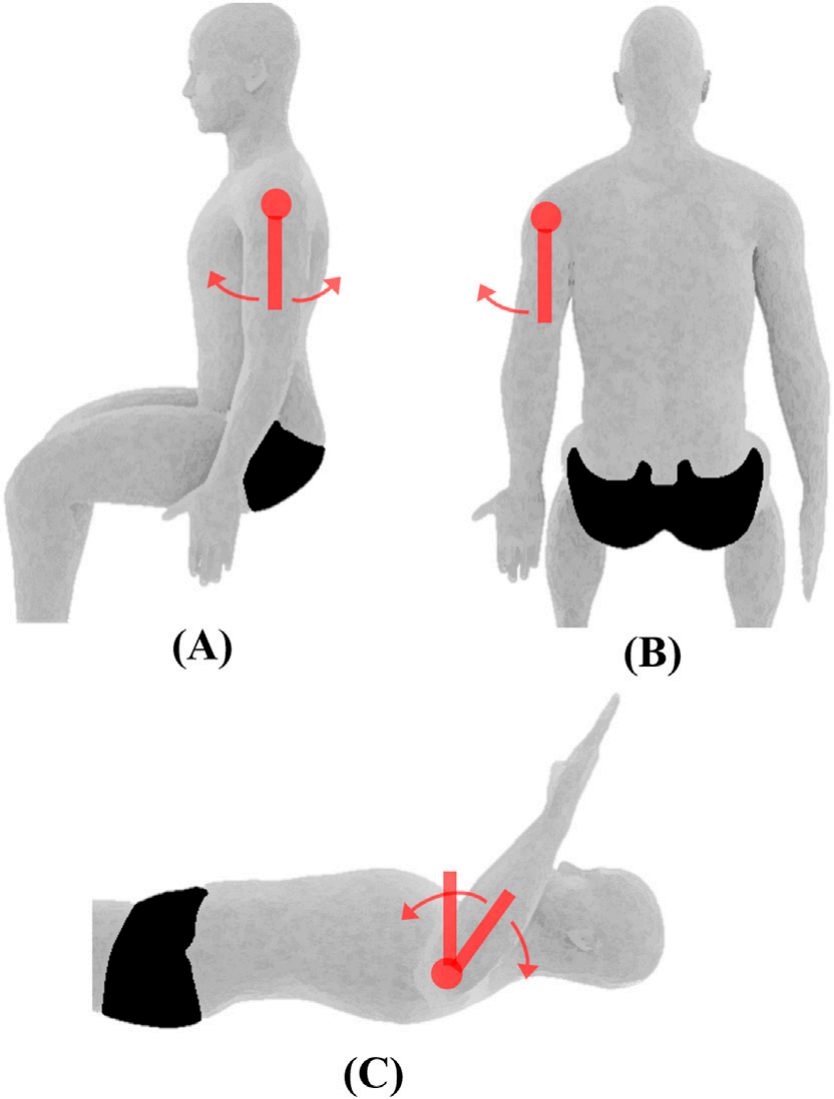
Shoulder joint ROM and initial position. **(A)** Flexion/extension, **(B)** abduction/adduction, **(C)** medial/lateral rotation. Measurements are performed using the universal goniometer (depicted by red swinging arms).

**FIGURE 2:**
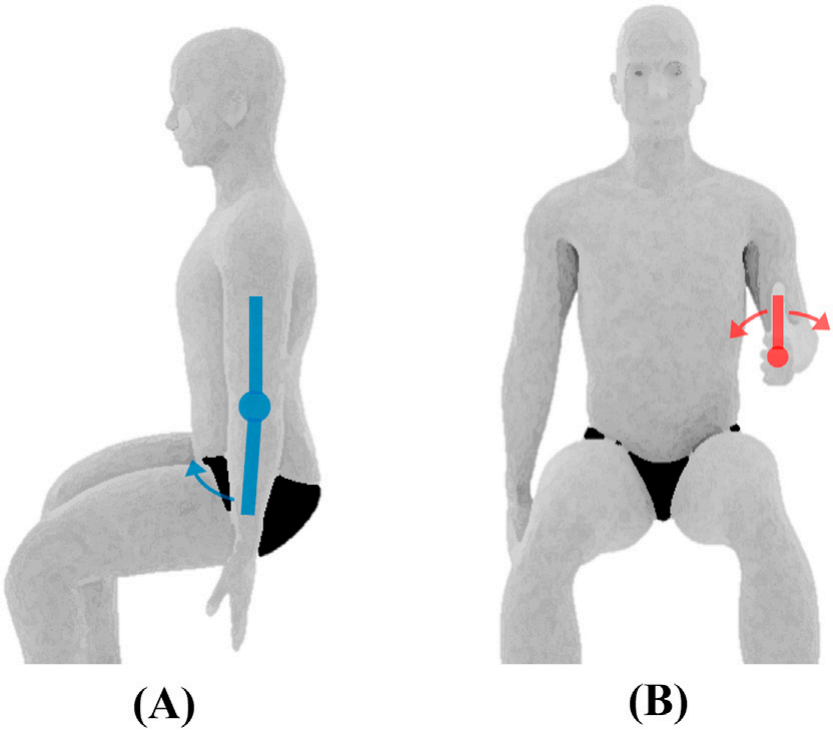
Elbow joint ROM and initial position. **(A)** Flexion/extension measured using the electric goniometer (depicted by the blue swinging arms), **(B)** pronation/supination of the forearm measured using the universal goniometer.

**FIGURE 3:**
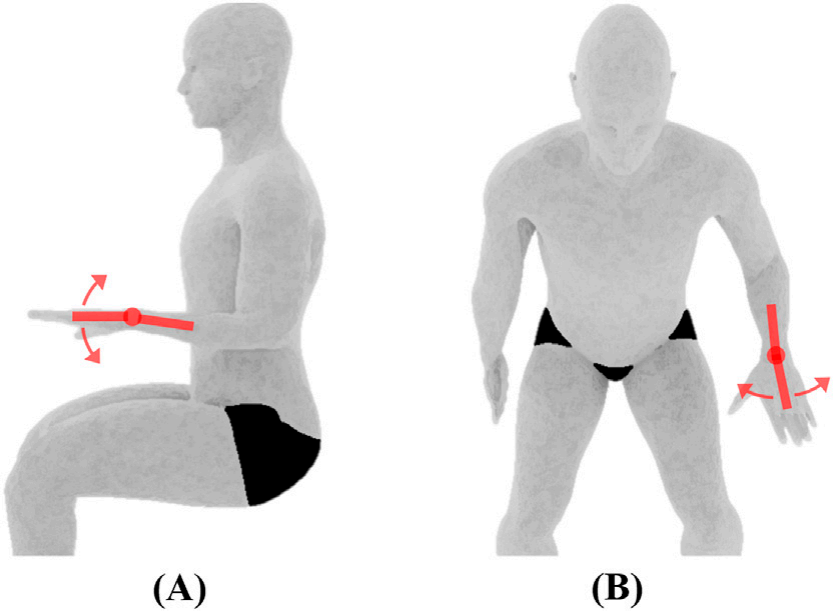
Wrist joint ROM and initial position. **(A)** Flexion/extension, **(B)** ulnar/radial deviation, both measured using the universal goniometer.

**TABLE 3:** Lower extremity joints ROM measurement procedures.

Motion		Starting position	Procedure
Hip	Flexion/Extension^a^	For flexion, the subject will be positioned supine with the hip and knee in anatomical position. For extension, the subject will be positioned prone with hip and knee in anatomical position (see Figures 4A,B)	The hip will be flexed by moving the femur anteriorly to the limit of the motion while allowing the knee to flex passively. The hip will be extended by moving the femur in the posterior direction until the limit of the motion
	Abduction/Adduction^a^	The subject will be positioned supine with the hip and knee in anatomical position (see Figure 4C)	The hip will be abducted by moving the femur laterally until the limit of motion. The hip will be adducted by moving the femur medially toward the contralateral limb. The hip will be maintained at 0° flexion/extension and 0° medial/lateral rotations
	Internal/External rotation^a^	The subject will be seated on a firm surface with his/her knees flexed to 90° over the edge of the surface (see Figure 4D)	For medial rotation, the tibia will be moved laterally away from the center of the body until the limit of the motion. For lateral rotation, the tibia will be moved medially toward the center of the body until the limit of the motion
Knee	Flexion/Extension^b^	The subject will be positioned supine with his/her knee and hip in anatomical position. A towel will be placed under the ankle to allow maximum knee extension. Knee extension will be recorded as the starting position for knee flexion (see Figure 5A)	The knee will be flexed by moving the heel toward the buttock until the limit of motion. The hip will also be flexed during this motion
	Internal/External Rotation^c^	The subject will lay on the side, with his/her knee flexed to 90°. The inclinometer will be placed on the lateral side of the foot (see Figure 5B)	The knee will be medially rotated by rotating the foot in the medial direction. The knee will be laterally rotated by rotating the foot in the lateral direction. The plantar surface of the foot will be maintained facing posteriorly
Ankle	Plantarflexion/Dorsiflexion^a^	The subject will be seated on a firm surface with his/her knees flexed to 90° over the edge of the surface. The ankle will be maintained hanging in the neutral position (see Figure 6A)	The ankle will be dorsiflexed by moving the dorsum of the foot upward toward the tibia. The ankle will be plantarflexed by moving the dorsum of the foot downward away from the tibia
	Inversion/Eversion^a^	The subject will be seated on a firm surface with his/her knees flexed to 90° over the edge of the surface. The ankle will be maintained in plantarflexion (see Figure 6B)	The ankle will be inverted by turning the foot inward such that the plantar surface of the foot is facing medially. The ankle will be everted by turning the foot outward such that the lateral side of the foot is higher than the medial side

^a^ Universal goniometer.

^b^ Electronic goniometer.

^c^ Inclinometer.

**FIGURE 4:**
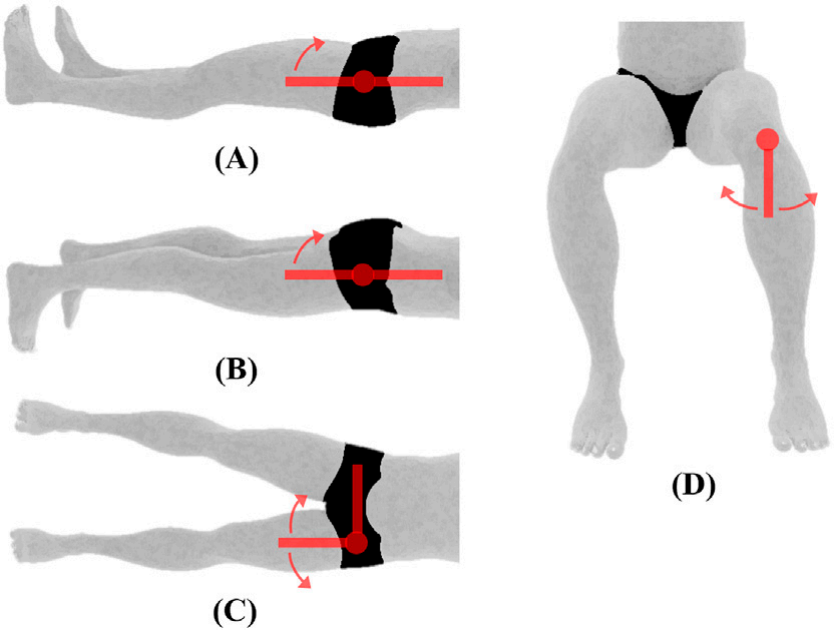
Hip joint ROM and initial position. **(A)** Flexion **(B)** extension, **(C)** abduction/adduction, **(D)** medial/lateral rotation, all measured using the universal goniometer.

**FIGURE 5:**
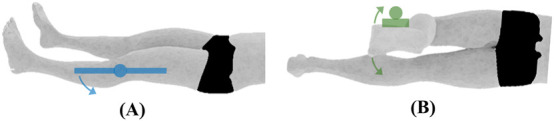
Knee joint ROM and initial position. **(A)** Flexion/extension measured using the electronic goniometer, **(B)** internal/external rotation measured using the inclinometer (depicted by the green shape).

**FIGURE 6:**
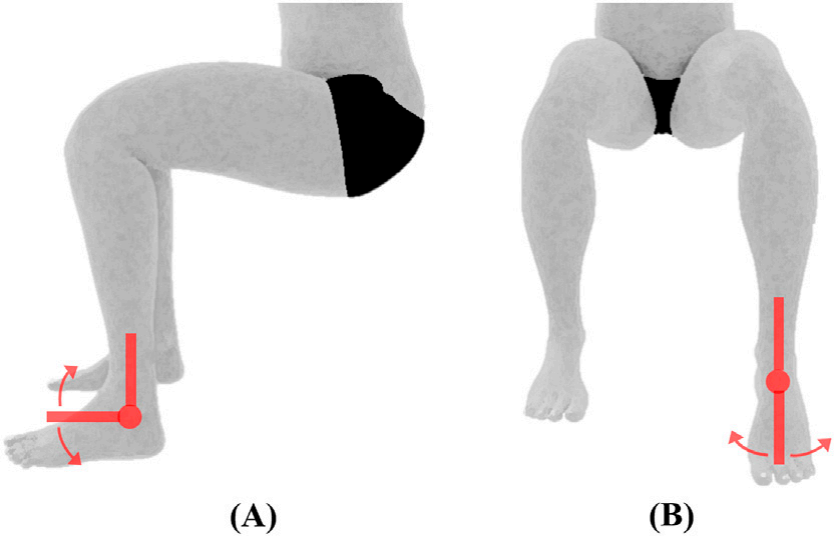
Ankle joint ROM and initial position. **(A)** Plantarflexion/dorsiflexion, **(B)** inversion/eversion, both measured using the universal goniometer.

### 3.2 Muscle strength and joint stiffness

Before the start of each testing session, the system will be calibrated to ensure the accuracy of the dynamometer measurements. The tests will be performed by trained researchers from UAEU. To measure the muscle strength, the dynamometer will be set to the isokinetic mode, which involves concentric contractions of the muscles to rotate the lever arm of the device at the set velocity. To measure the joint stiffness, the dynamometer will be set to the reactive eccentric mode which involves eccentric contractions to resist the rotation of the lever arm at the set velocity. The muscle strength test will precede the joint stiffness test.

A general warm up for the whole group (n = 10) will be performed before testing, which will include 20-s jog in place, 10 jumping jacks, 10 high-knees and 10 arm circles. Specific warm up will be then performed prior to each test for each participant, which will include three submaximal and one maximal repetition of the designated motion. This will allow for the familiarization of the motion while reducing the potential for injuries during the maximal effort test. A 30-s rest will be given before proceeding with the maximal effort tests. For muscle strength assessment, three repetitions of maximal effort concentric contractions will be performed at two different velocities specific to each joint. The test velocities will be separated by a minute rest. A 2-min rest will be allowed upon completing the concentric tests which will be followed by the eccentric (reactive) tests. For joint stiffness assessment, three repetitions of maximal effort eccentric contractions will be performed at a single velocity specific to each joint. The participants will be carefully instructed on how to perform eccentric contractions by manually guiding them through the movement. The muscle strength and joint stiffness assessments of each joint motion for one body side will be conducted for all 10 children before readjusting the dynamometer to assess the contralateral side. This procedure will be repeated for all joints and motions. The ROM of the dynamometer will be adjusted for every participant before testing.

**FIGURE 7:**
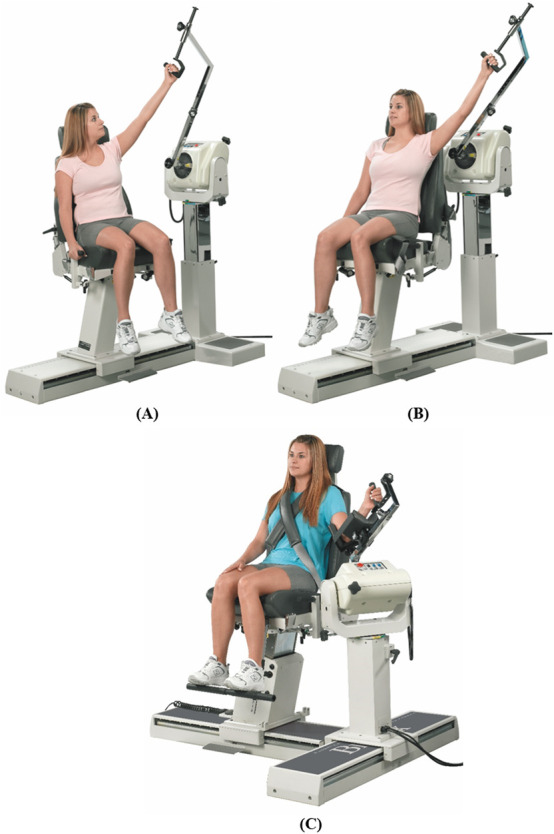
Dynamometer and seat adjustment for shoulder joint rotations. **(A)** Flexion/extension, **(B)** abduction/adduction, **(C)** medial/lateral rotation (reproduced with permission from Biodex).

**FIGURE 8:**
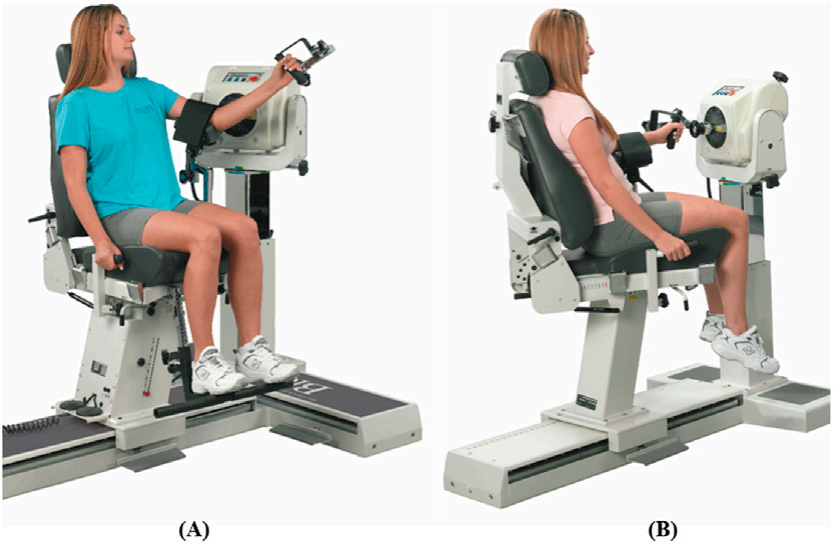
Dynamometer and seat adjustment for elbow joint rotations. **(A)** Flexion/extension, **(B)** pronation/supination of the forearm (reproduced with permission from Biodex).

**FIGURE 9:**
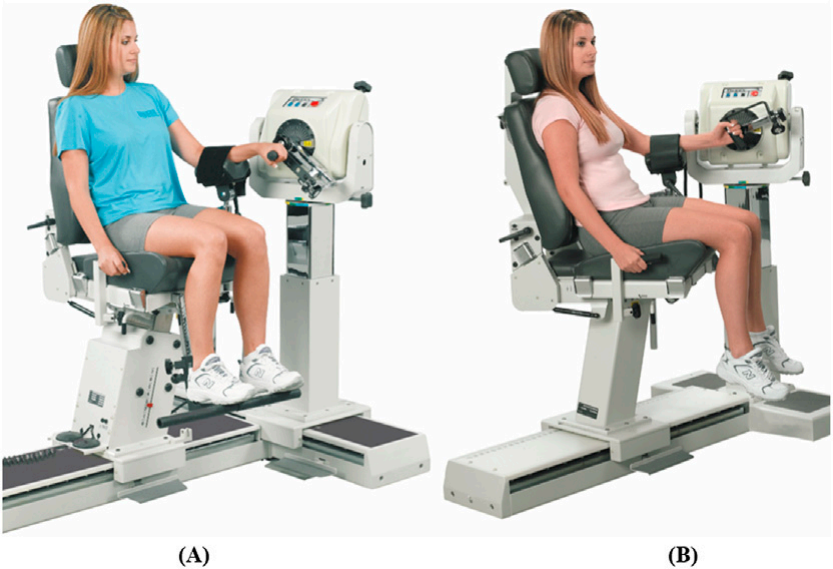
Dynamometer and seat adjustment for wrist joint rotations. **(A)** Flexion/extension, **(B)** radial/ulnar deviation (reproduced with permission from Biodex).

**FIGURE 10:**
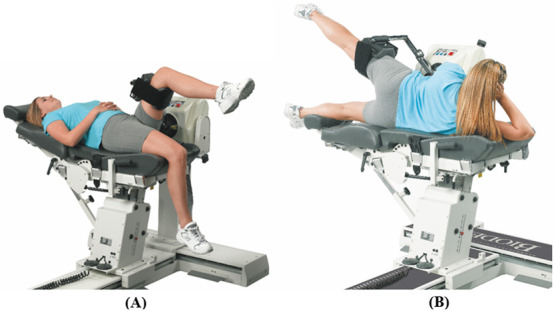
Dynamometer and seat adjustment for hip joint rotations. **(A)** Flexion/extension, **(B)** abduction/adduction (reproduced with permission from Biodex).

**FIGURE 11:**
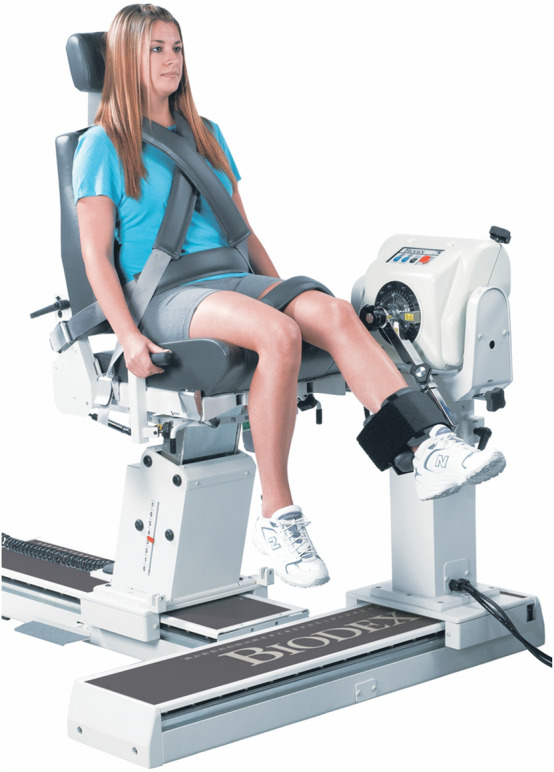
Dynamometer and seat adjustment for knee flexion/extension (reproduced with permission from Biodex).

**FIGURE 12:**
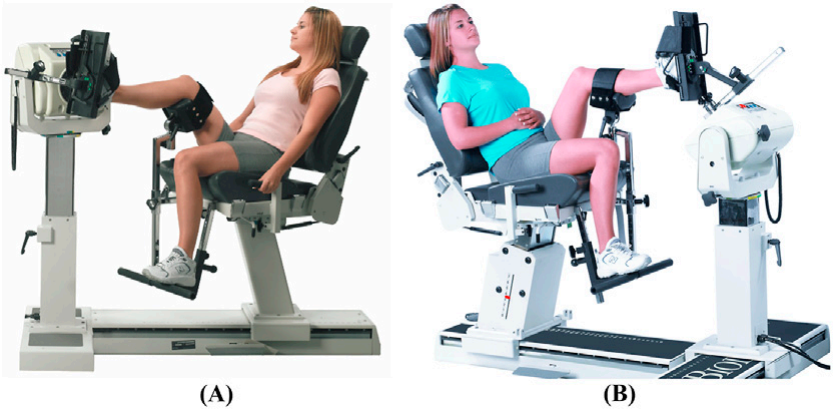
Dynamometer and seat adjustment for ankle rotations. **(A)** Plantarflexion/dorsiflexion, **(B)** inversion/eversion (reproduced with permission from Biodex).

Given the small body sizes of some of the participating children, their muscles may not be sufficiently developed to perform isokinetic testing through the full ROM of certain joints. For children unable to perform the full ROM, the ROM will be set from the point where they are able to move the dynamometer arm. This will be determined by manually assisting the child until the point in the ROM where he or she is able to move the dynamometer arm. If they are entirely unable to move the dynamometer arm at any point in the ROM for a given movement, only eccentric contractions will be performed where the device moves automatically, and the child resists the motion. Additionally, research highlights the importance of learning effects when assessing children (Hayes and Petersen, 2001; Fagher et al., 2016). This emphasizes the need for pre-test familiarization of the designated movement. The movements will be demonstrated through pictures and verbal instructions. Participants will be given verbal and visual feedback through the on-screen display. Although there has been no publications highlighting the significance of verbal encouragement on the pediatric population (De et al., 2003), it has been suggested that children perform better when they are given visual feedback (Baltzopoulos and Kellis, 1998). Incorporating enjoyable activities and the use of verbal encouragement to develop a sense of success have been reported as facilitators for physical participation among children (Antoniadou et al., 2024). For joint stiffness assessment, the children will be carefully instructed on how to perform eccentric contractions by manually guiding them through the movement. The following subsections will describe, in detail, the joint-specific settings and procedures for the testing process (refer to Figures 7–12).

#### 3.2.1 Shoulder

Shoulder rotations will be performed in a seated position with the seatback tilted to 85°, and the participant will be stabilized by attaching the shoulder and waist straps. The velocity of the dynamometer will be set at 60° and 90°/s for concentric contraction tests and at 60°/s for the eccentric contraction test.

##### 3.2.1.1 Flexion/extension

Rotation occurs in the sagittal plane about an approximation of the line connecting the acromion to the trigonum spinae. The dynamometer shaft will be aligned with the axis of rotation by pointing it towards the acromion at a distance of approximately 3 cm. For the starting position, the shoulder will be kept in full extension with 0° abduction/adduction and internal/external rotation (see Figure 7A). The elbow will be slightly flexed to avoid potential injury from excessive loading on the elbow joint. The participant will rotate the lever arm away from the body (shoulder flexion) with maximal effort until the end of the pre-set ROM. The lever arm will then be rotated back to the starting position (shoulder extension) to complete one cycle.

##### 3.2.1.2 Abduction/adduction

Rotation occurs in the frontal plane about an approximation of the line pointing anteriorly perpendicular to an extending line from the midpoint of the lateral-medial epicondyles of the humerus to the glenohumeral joint. The dynamometer shaft will be aligned with the axis of rotation by pointing it towards the acromion at a distance of approximately 3 cm. For the starting position, the shoulder will be kept in full adduction with 0° flexion/extension and slight shoulder external rotation for the participant’s comfort (see Figure 7B). The elbow will be slightly flexed to avoid potential injury from excessive loading on the elbow joint. The participant will rotate the lever arm away from the body (shoulder abduction) with maximal effort until the end of the pre-set ROM. The lever arm will then be rotated back to the starting position (shoulder adduction) to complete one cycle.

##### 3.2.1.3 Internal/external rotation

Rotation occurs in the sagittal plane about an approximation of the line pointing laterally from the glenohumeral joint. The dynamometer shaft will be aligned with the axis of rotation by pointing it towards the acromion at a distance of approximately 3 cm. For the starting position, the shoulder will be kept in full internal rotation with 40°–50° abduction of the shoulder (dynamometer tilted at the angle of shoulder abduction) and elbow at 90° flexion (see Figure 7C). The participant will rotate the lever arm upwards away from the body (shoulder external rotation) with maximal effort until the end of the pre-set ROM. The lever arm will then be rotated back to the starting position (shoulder internal rotation) to complete one cycle.

#### 3.2.2 Elbow

Elbow rotations will be performed in a seated position with the seatback tilted to 70°–85° and the participant will be stabilized by attaching the shoulder and waist straps. The elbow will be supported using the limb-support pad. The velocity of the dynamometer will be set at 60° and 90°/s for concentric contraction tests and at 60°/s for the eccentric contraction test.

##### 3.2.2.1 Flexion/extension

Rotation occurs in the sagittal plane about an approximation of the line connecting the lateral and medial epicondyles of the humerus. The dynamometer shaft will be aligned with the axis of rotation by pointing it towards the lateral epicondyle of the humerus at a distance of approximately 3 cm. For the starting position, the elbow will be kept in full flexion with approximately 45° shoulder flexion (see Figure 8A). The handgrip may rotate freely during the motion. The participant will rotate the lever arm away from the body (elbow extension) with maximal effort until the end of the pre-set ROM. The lever arm will then be rotated back to the starting position (elbow flexion) to complete one cycle.

##### 3.2.2.2 Pronation/supination of the forearm

Rotation occurs in the frontal plane about an approximation of the line extending from the midpoint of the lateral-medial epicondyles of the humerus to the midpoint of the ulnar-radial styloid. The dynamometer shaft will be aligned with the axis of rotation by pointing it towards the midpoint of the ulnar-radial styloid at a distance of approximately 3 cm. For the starting position, the forearm will be kept in full pronation with elbow in 90° flexion and slight flexion of the shoulder (see Figure 8B). The participant will rotate the lever arm away from the body (forearm supination) with maximal effort until the end of the pre-set ROM. The lever arm will then be rotated back to the starting position (forearm pronation) to complete one cycle.

#### 3.2.3 Wrist

Wrist rotations will be performed in a seated position with the seatback tilted to 85° and the participant will be stabilized by attaching the shoulder and waist straps. The elbow will be supported using the limb-support pad. The velocity of the dynamometer will be set at 30° and 60°/s for concentric contraction tests and at 30°/s for the eccentric contraction test.

##### 3.2.3.1 Flexion/extension

Rotation occurs in the sagittal plane about an approximation of the line connecting the radial and ulnar styloid. The dynamometer shaft will be aligned with the axis of rotation by pointing it towards the ulnar styloid at a distance of approximately 3 cm. For the starting position, the wrist will be kept in full flexion with the shoulder and elbow at approximately 45° flexion (see Figure 9A). The participant will rotate the lever arm away from the body (wrist extension) with maximal effort until the end of the pre-set ROM. The lever arm will then be rotated back to the starting position (wrist flexion) to complete one cycle.

##### 3.2.3.2 Ulnar/radial deviation

Rotation occurs in the sagittal plane about an approximation of the line pointing laterally perpendicular to the midpoint of the radial and ulnar styloid. The dynamometer shaft will be aligned with the axis of rotation by pointing it towards the midpoint of the ulnar-radial styloid at a distance of approximately 3 cm. For the starting position, the wrist will be kept in full ulnar deviation with the forearm at 0° pronation/supination and the shoulder and elbow at approximately 45° flexion (see Figure 9B). The participant will rotate the lever arm away from the body (radial deviation) with maximal effort until the end of the pre-set ROM. The lever arm will then be rotated back to the starting position (ulnar deviation) to complete one cycle.

#### 3.2.4 Hip

The seatback will be fully reclined for the hip rotations. Hip flexion/extension motion will be performed in a supine position and abduction/adduction performed having the participant lying on his/her side. The hip attachment will be placed slightly superior to the popliteal fossa. The velocity of the dynamometer will be set at 60° and 90°/s for concentric contraction tests and at 60°/s for the eccentric contraction test.

##### 3.2.4.1 Flexion/extension

Rotation occurs in the sagittal plane about an approximation of the line pointing laterally from the greater trochanter. The dynamometer shaft will be aligned with the axis of rotation by pointing it towards the greater trochanter at a distance of approximately 3 cm. For the starting position, the hip will be kept in full extension with the knee at approximately 45° flexion (see Figure 10A). The participant will rotate the lever arm away from the body (hip flexion) with maximal effort until the end of the pre-set ROM. The lever arm will then be rotated back to the starting position (hip extension) to complete one cycle.

##### 3.2.4.2 Abduction/adduction

Rotation occurs in the frontal plane about an approximation of the line pointing anteriorly from the greater trochanter. The dynamometer shaft will be aligned with the axis of rotation by pointing it towards the greater trochanter at a distance of approximately 3 cm. For the starting position, the hip will be kept in full adduction with hip flexion/extension at 0° (see Figure 10B). The participant will rotate the lever arm away from the body (hip abduction) with maximal effort until the end of the pre-set ROM. The lever arm will then be rotated back to the starting position (hip adduction) to complete one cycle.

#### 3.2.5 Knee

The motion will be performed in a seated position with the seatback tilted to 70°–85° depending on the comfort of participant. The participant will be stabilized with shoulder, waist, and thigh straps. The knee attachment will be placed slightly superior to the medial and lateral malleoli. The velocity of the dynamometer will be set at 60° and 90°/s for concentric contraction tests and at 60°/s for the eccentric contraction test.

##### 3.2.5.1 Flexion/extension

Rotation occurs in the sagittal plane about the line extending from the femoral lateral epicondyle to the medial epicondyle. The dynamometer shaft will be aligned with the axis of rotation by pointing it towards the lateral epicondyle of the femur at a distance of approximately 3 cm. For the starting position, the knee will be kept in full flexion (see Figure 11). The participant will rotate the lever arm away from the body (knee extension) with maximal effort until the end of the pre-set ROM. The lever arm will then be rotated back to the starting position (knee flexion) to complete one cycle.

#### 3.2.6 Ankle

Ankle rotations will be performed in a seated position with the seatback tilted to 55°–70° and shoulder and waist straps attached to stabilize the participant. The thigh will be supported by placing the limb-support pad under the distal femur. A cushion may be placed over the dorsum of the foot to ensure close-fitting of the ankle attachment. The velocity of the dynamometer will be set at 60° and 90°/s for concentric contraction tests and at 60°/s for the eccentric contraction test.

##### 3.2.6.1 Dorsiflexion/plantarflexion

Rotation occurs in the sagittal plane about the line connecting the lateral and medial malleoli. The dynamometer shaft will be aligned with the axis of rotation by pointing it towards the lateral malleolus at a distance of approximately 3 cm. For the starting position, the ankle will be kept in full plantarflexion with the knee at approximately 45° flexion (see Figure 12A). The participant will rotate the lever arm away from the body (ankle dorsiflexion) with maximal effort until the end of the pre-set ROM. The lever arm will then be rotated back to the starting position (ankle plantarflexion) to complete one cycle.

##### 3.2.6.2 Inversion/eversion

Rotation occurs in the frontal plane about the line extending from the midpoint of the lateral and medial malleoli to the calcaneus at approximately 45°. The dynamometer shaft will be aligned with the axis of rotation by pointing it towards the calcaneus at approximately 45°. For the starting position, the ankle will be kept in full inversion with the knee at approximately 45° flexion (see Figure 12B). The participant will rotate the lever arm away from the body (ankle eversion) with maximal effort until the end of the pre-set ROM. The lever arm will then be rotated back to the starting position (ankle inversion) to complete one cycle.

## 4 Anticipated results

The ROM for each joint motion will be defined by the maximum angular displacements between the flexed and extended positions. For example, the elbow joint ROM will be represented by the angles of maximum flexion and maximum extension.

The muscle strength will be represented by the peak torque produced by the muscle group responsible for each joint motion, with a single maximum value extracted from the torque-angle curve generated by the Biodex system. The joint stiffness will be calculated as the gradient of the torque-angle curve.

The data collected will be securely stored on a computer. The sample of 200 participants will be divided into strata based on three key variables: ethnicity, age and gender. The participants will be grouped into three major ethnic groups: South Asian, Arab and Others. Gender will be grouped into two (boys and girls) and age will be grouped into nine categories (ages 4–12). Each stratum will have an equal number of participants for a proportional representation across all groups.

Normality check will be performed for the data obtained using the Kolmogorov-Smirnov test and parametric or non-parametric tests will be employed accordingly. Descriptive statistical analyses will be conducted to summarize the three parameters investigated in this study. The relationship between categorical variables will be analyzed using either Pearson’s Chi-squared test or Fisher’s exact test as appropriate. Similarly, the association between categorical and numeric variables will be examined using either Mann-Whitney U test or independent samples t-test while the relationship between numeric variables will be analyzed using either Pearson or Spearman correlation analysis. To determine the influence of anthropometric (body mass, height, waist circumference, etc.) and demographic (gender, age, and ethnicity) factors on ROM, strength, and stiffness, a series of multiple regression analyses will be performed to identify the factors that can best predict ROM, strength, and stiffness. Significance level will be set at 5%.

## 5 Discussion

This protocol describes a cross-sectional study aimed at establishing reference values for joint ROM, muscle strength, and joint stiffness. It should be noted that due to the cross-sectional study design, causal relationships between the measured variables cannot be determined. Nevertheless, the findings will provide valuable baseline evidence and inform the design of future longitudinal or interventional studies. The protocol was developed to enable comprehensive assessments of the pediatric upper and lower extremity joints (shoulder, elbow, wrist, hip, knee, and ankle) function focusing on the evaluation of the joint ROM, joint stiffness, and the strength of the surrounding muscles. True stiffness of the human joint can be regarded as the combination of all the individual stiffness values contributed by muscles, tendons, ligaments, cartilages, and bones (Latash and Zatsiorsky, 1993). However, it has been acknowledged that the mathematical expressions to model all the components that contribute to a certain motion have not been developed yet (Butler et al., 2003). While some muscles contribute to a motion by generating a force to stabilize the joint (Escamilla et al., 2009), only the primary (agonistic) muscles which create the movement will be included in the muscle groups under assessment in the proposed study. The primary muscles involved in the specific motions were identified through studies that investigated these muscles (Refer to Supplementary Appendix 1). Isokinetic dynamometry enables the measurement of muscle strength and joint stiffness in both children and adults. The Biodex System 4 allows for the adjustment of the seat and dynamometer heights and orientations to accommodate the different limb lengths of subjects. When examining muscle function in children, it is necessary to consider the gravitational torques that contribute to the motion during testing. Movements in the sagittal and the frontal plane will be either assisted by gravity (e.g., knee flexion) or resisted by gravity (e.g., knee extension). This is particularly important when assessing children as they produce lower muscle torque compared to adults, leading to a higher percentage error (Jones and Stratton, 2000). The Biodex System 4 offers a gravity correcting feature which adjusts the additional torque generated due to gravity.

Several studies have attempted to draw a correlation between extremity joint muscle strength with demographic and anthropometric characteristics. For example, a study by Danneskiold‐Samsøe et al. (2009) investigated the dependency of muscle strength of the upper and lower extremities on gender, age, height, weight and BMI for an adult population while Forthomme et al. (2002) investigated the effects of weight, limb dominance and gender on the strength of forearm pronators and supinators, and wrist flexors and extensors. In addition, Marcolin et al. (2009) assessed the strength of several muscles in children with growing pains and joint hypermobility to make comparisons with healthy children. Wiggin et al. (2006) developed a protocol to establish percentile charts of isokinetic peak torque strength for the quadriceps and hamstrings by gender and age. Thus, the protocol presented in the current study will facilitate the examination of the muscle strength and joint stiffness of the upper and lower extremities of healthy children and correlate these factors with demographic and anthropometric variables. Moreover, the ROM will be correlated with the muscle strength and joint stiffness among healthy children to explore the relation between these variables. A study by Kalkman et al. (2019) identified a correlation between tendon stiffness and limited joint ROM among children with cerebral palsy, suggesting that these correlations may vary across different pediatric populations.

Injuries to both the upper and lower extremities are the second most common types of injuries sustained by children in vehicle crashes globally (Hanna, 2010; Arbogast et al., 2002) with joint kinematics and joint stiffness playing a key role in determining the severity of subsequent injuries (Seidel et al., 2018; Fildes et al., 1997; Boucher et al., 2017).

Understanding the correlation between the biomechanical factors (ROM, muscle strength and joint stiffness) of the upper and lower extremities, and the demographic and anthropometric characteristics is essential for the development of child vehicle safety systems. Research suggests that current standards for evaluating extremity injuries in pediatric crash tests remain limited, as child ATDs (anthropometric test devices) lack adequate instrumentation to measure upper and lower limb injuries (Boucher et al., 2016; Boucher, 2014; Boucher et al., 2017; Boucher et al., 2013). The proposed study will provide essential data to quantify biomechanical tolerance in children and support the development of more biofidelic extremities for child ATDs and computational models, ultimately contributing to more effective pediatric restraint systems. Measuring the pediatric biomechanical tolerances are therefore valuable for improving both injury prediction and child vehicle safety systems. In addition, this data can support clinical applications beyond vehicle safety. It has been suggested that muscle strength in pathological populations can be improved through interventions such as resistance training (Hanssen et al., 2022; Liu et al., 2025), thus an understanding of the biomechanical characteristics of healthy populations is not only valuable for diagnosis, but also for measuring the efficacy of interventions. Moreover, the extremity joints ROM of children with DS are limited compared to typically developing children, which emphasizes the importance of having baseline data from healthy children to guide diagnosis and rehabilitation (Jones et al., 2023). The proposed study will provide normative data that can serve as a reference for clinical assessment and rehabilitation planning.

Abdulazeez et al. (2024), Abdulazeez et al. (2025) conducted a large-scale cross-sectional study on healthy children (1–12 years) demonstrating that knee and elbow ROM varies with anthropometric and demographic factors. Building on this knowledge, our study will extend the scope to include upper and lower extremity joints. Similarly, Scherff et al. (2024) examined the ROM and muscle strength of typically developing children and suggested that future studies should investigate the gender differences across different age groups. In addition, González-Matilla et al. (2025) compared the ROM of the lower extremity joints of healthy children and children with CP, which concluded that children with CP exhibit reduced ROM. However, it was reported that the limited sample size restricted the extrapolation of the results. To the best of our knowledge, only a limited number of studies have assessed muscle strength and joint stiffness in pediatric extremity joints, with most of the recent studies focusing on children with neuromuscular diseases (NMDs). Van Tittelboom et al. (2022) noted that their study was limited by the small sample size. They also reported that the variation between test protocols makes comparison difficult between studies, highlighting the need for standardized protocols (e.g., participant positioning, testing velocities, rest time). Our protocol closely aligns with procedures reported by Van Tittelboom et al. More recently, van der Woude et al. highlighted the usefulness of cutoff values (minimum threshold) to decide whether children with NMDs are strong enough to perform isokinetic testing (van der Woude et al., 2024). In line with this, our study will present the cutoff values in terms of the dynamometer ROM in relation to age or anthropometric factors to determine at what point healthy children are able to perform concentric contractions.

Research on muscle strength and joint stiffness has largely focused on the adult population with the limited number of studies examining pediatric extremity joint and muscle function mostly focused on children with disabilities such as cerebral palsy and Down syndrome. Additionally, most of these studies have concentrated on lower extremity joints only particularly the knee joint. This protocol offers a foundation for future research for conducting comprehensive assessments of the pediatric extremity joint function for measuring the ROM, muscle strength and joint stiffness in pediatric extremity joints (shoulder, elbow, wrist, hip, knee, and ankle) using goniometry and isokinetic dynamometry. Future studies investigating pediatric injury mechanisms and impact forces in vehicle collisions should incorporate extremity biomechanical data in their ATDs and computational models for more accurate evaluation of injury risk. In addition, it would be beneficial to investigate the biomechanical characteristics of pathological populations, such as children with CP or DS, using the same measurement procedures as in healthy children, to allow for comparison between the different populations.

While our proposed study will provide baseline ROM, muscle strength, and joint stiffness measures in typically developing children, pathological populations such as children with CP or DS often demonstrate reduced ROM, lower muscle strength and altered joint stiffness (González-Matilla et al., 2025; Koo et al., 2022; Leister et al., 2024). Therefore, the findings from this study cannot be generalized to children with pathological conditions. Additionally, due to the limited isokinetic dynamometer settings allowing only one configuration per joint, variations in the number of sets, repetitions, machine velocity, etc., to determine the optimal conditions for peak performance in children cannot be explored.

## Data Availability

The original contributions presented in the study are included in the article/Supplementary Material, further inquiries can be directed to the corresponding author.
