# Supplementary material for: Evaluation of joint and muscle function of paediatric upper and lower extremities
Source: Front Bioeng Biotechnol. 2025 Oct 29;13:1667865. doi: 10.3389/fbioe.2025.1667865 (PMC12605018; doi:10.3389/fbioe.2025.1667865)
Supplement: Supplementary file 1 [file Presentation1.pdf]

Appendix 1. Muscle groups involved

Table 1 – Muscle groups involved in each joint movement.

Joint	Muscle Group	Muscles Involved
**Shoulder**	Flexor	Deltoid, Supraspinatus, Infraspinatus, Subscapularis
	Extensor	Posterior Deltoid, Latissimus Dorsi, Teres Major
	Abductor	Supraspinatus, Deltoid, Infraspinatus, Subscapularis
	Adductor	Teres Major, Latissimus Dorsi, Pectoralis Major
	Internal Rotator	Anterior Deltoid, Subscapularis, Pectoralis Major
	External Rotator	Deltoid, Supraspinatus, Infraspinatus, Subscapularis, Teres Minor
**Elbow**	Flexor	Biceps Brachii, Brachialis, Brachioradialis
	Extensor	Triceps Brachii
	Forearm Pronator	Pronator Teres, Pronator Quadratus
	Forearm Supinator	Biceps Brachii, Supinator
**Wrist**	Flexor	Flexor Carpi Ulnaris, Flexor Carpi Radialis
	Extensor	Extensor Carpi Ulnaris, Extensor Carpi Radialis, Extensor Digitorum
	Ulnar Deviator	Flexor Carpi Ulnaris, Extensor Carpi Ulnaris
	Radial Deviator	Flexor Carpi Radialis, Extensor Carpi Radialis
**Hip**	Flexor	Iliacus, Psoas Major, Rectus Femoris, Sartorius, Tensor Fascia Latae, Rectus Abdominis, Obliquus Externus, Obliquus Internus
	Extensor	Gluteus Maximus, Semitendinosus, Semimembranosus, Biceps Femoris, Adductor Magnus, Erector Spinae, Multifidus
	Abductor	Gluteus Medius, Gluteus Minimus, Tensor Fascia Latae, Sartorius
	Adductor	Adductor Brevis, Adductor Longus, Adductor Magnus, Gracilis
**Knee**	Flexor	Rectus Femoris, Vastus Lateralis, Vastus Medialis, Vastus Intermedius
	Extensor	Biceps Femoris, Semitendinosus, Semimembranosus, Sartorius, Gracilis
	Internal Rotator	Semitendinosus, Semimembranosus, Sartorius, Gracilis
	External Rotator	Biceps Femoris
**Ankle**	Plantarflexor	Gastrocnemius Medialis, Gastrocnemius Lateralis, Soleus, Fibularis Longus, Flexor Digitorum Longus, Flexor Hallucis Longus, Tibialis Posterior
	Dorsiflexor	Tibialis Anterior, Extensor Digitorum Longus
	Invertor	Tibialis Anterior, Tibialis Posterior
	Evertor	Fibularis Longus, Fibularis Brevis

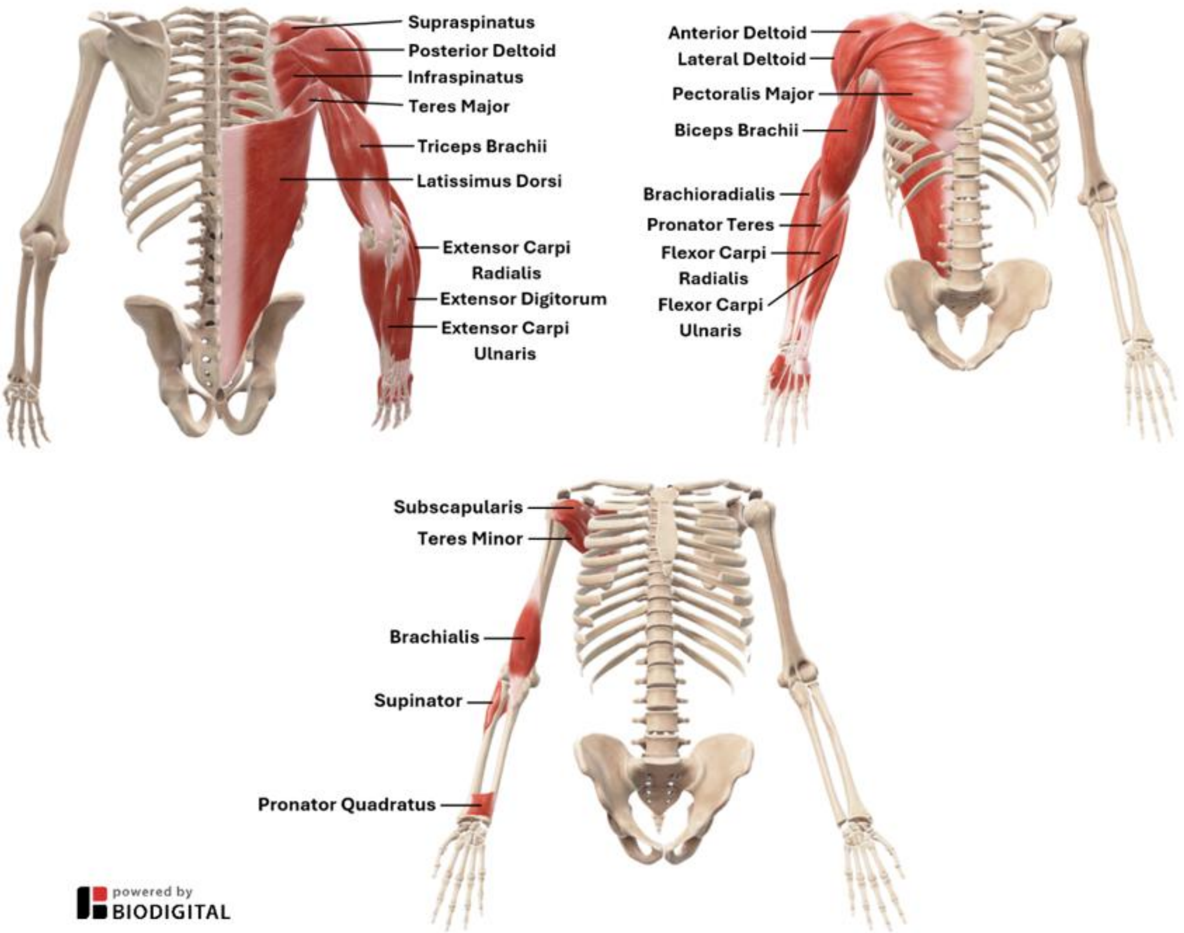

powered by BIODIGITAL

Figure 1 – Upper extremity muscles involved (posterior, anterior, deep muscles).

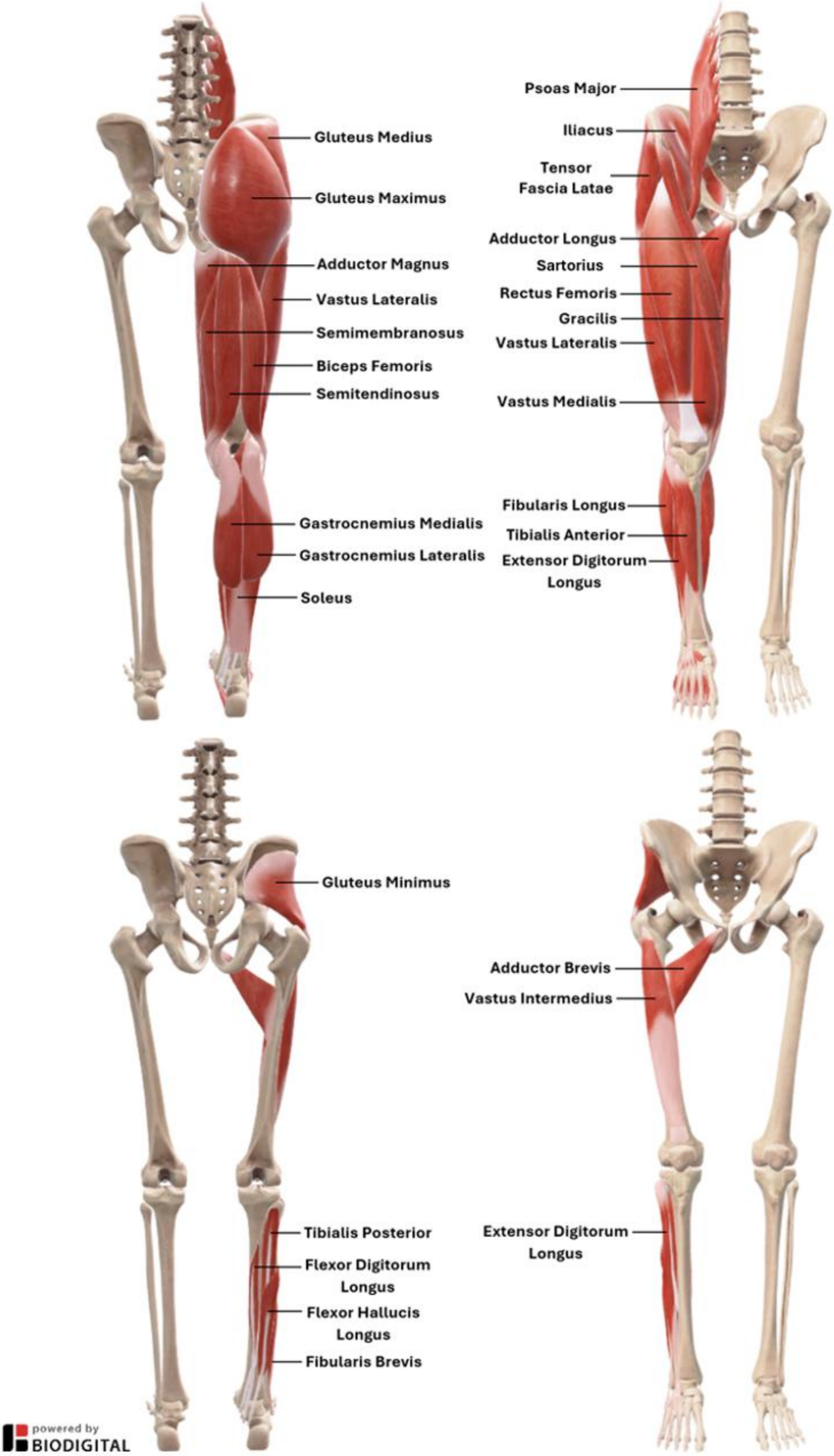

Figure 2 – Lower extremity muscles involved (posterior, anterior, deep muscles).
